# Surface Defect Detection System for Carrot Combine Harvest Based on Multi-Stage Knowledge Distillation

**DOI:** 10.3390/foods12040793

**Published:** 2023-02-13

**Authors:** Wenqi Zhou, Chao Song, Kai Song, Nuan Wen, Xiaobo Sun, Pengxiang Gao

**Affiliations:** College of Engineering, Northeast Agricultural University, Harbin 150030, China

**Keywords:** carrot, surface defect detection and sorting, multi-stage knowledge distillation network, network lightweight

## Abstract

Carrots are a type of vegetable with high nutrition. Before entering the market, the surface defect detection and sorting of carrots can greatly improve food safety and quality. To detect defects on the surfaces of carrots during combine harvest stage, this study proposed an improved knowledge distillation network structure that took yolo-v5s as the teacher network and a lightweight network that replaced the backbone network with mobilenetv2 and completed channel pruning as a student network (mobile-slimv5s). To make the improved student network adapt to the image blur caused by the vibration of the carrot combine harvester, we put the ordinary dataset Dataset (T) and dataset Dataset (S), which contains motion blurring treatment, into the teacher network and the improved lightweight network, respectively, for learning. By connecting multi-stage features of the teacher network, knowledge distillation was carried out, and different weight values were set for each feature to realize that the multi-stage features of the teacher network guide the single-layer output of the student network. Finally, the optimal lightweight network mobile-slimv5s was established, with a network model size of 5.37 MB. The experimental results show that when the learning rate is set to 0.0001, the batch size is set to 64, and the dropout is set to 0.65, the model accuracy of mobile-slimv5s is 90.7%, which is significantly higher than other algorithms. It can synchronously realize carrot harvesting and surface defect detection. This study laid a theoretical foundation for applying knowledge distillation structures to the simultaneous operations of crop combine harvesting and surface defect detection in a field environment. This study effectively improves the accuracy of crop sorting in the field and contributes to the development of smart agriculture.

## 1. Introduction

Carrots are one of the top ten vegetable crops in the world and is popularized for its easy planting mode and high nutrition [1]. According to the statistics of the Food and Agriculture Organization of the United Nations, China planted about 470,000 hectares of carrots in 2021, which is the largest carrot planting country in the world [2]. During the growth and harvesting of carrots, there will be bifurcation, deformation, cracking, and other deformable phenomena due to the influence of natural factors like weather, insect pests, and the planting environment [3]. At present, the carrot harvesting method is gradually shifting towards combine harvesting. The phenomenon of carrot breakage is easy to occur during combine harvesting because of human factors such as improper machine operation and the mismatch of agronomic planting requirements. The existence of defective carrots will lead to storage difficulties and rotting, which would cause serious food safety problems if not sorted. Therefore, carrot defect detection is the most important step after carrot picking [4]. It is of great economic value to select the modes of transportation and sales according to carrot defect detection and sorting [5]. Currently, carrot surface defect detection and sorting are mostly performed manually or are sorted at the post-processing stage at the factory, resulting in high operation costs and low efficiency [1].

Therefore, it is essential to develop a surface defect detection system suitable for carrot combine harvesters by means of a computer neural network algorithm [6]. Simultaneous operation of crop combine harvesting and surface detection sorting has been fully applied in crop harvesting [7]. Calixto RR et al. used two classifiers (KNN or MLP) to realize real-time sorting of melons during field harvesting through machine vision nondestructive testing technology to avoid bringing back melons of poor quality to the packaging factory or the market, which may cause food safety incidents [8]. Ni Hongjun et al. realized surface defect analysis, identification, and sorting of winter jujube in the field through a BP (back propagation) neural network and used a winter jujube sorting mechanism to achieve the classification of red jujube. They proved that this method could realize the integration of winter-jujube combine harvesting and sorting in the field, and the sorting accuracy was more than 90% [9].

In recent years, machine learning and image processing technology have been widely used in crop harvesting and sorting. The objective and nondestructive detection of crops using computer vision systems has been proven to be successful in different operating environments. Zou et al. adopted the method of three cameras to obtain fruit surface color characteristics and used the threshold segmentation method to extract ROI from the image region of interest so as to realize fruit defect sorting. However, the accuracy of this characteristic sorting method is not high, and the computation amount is high [10]. J. lasco et al. designed a region-oriented segmentation algorithm to detect the most common peel defects in citrus fruits. This method can identify minor flaws of different varieties of oranges and citrus, with a recognition accuracy of greater than 95% [11]. Liming and Yanchao et al. classified strawberry images by drawing lines, which can effectively obtain the shape feature [12]. Machine learning has also been applied to the detection and sorting of carrot appearances. Han et al. suggested using machine learning to identify and sort carrot appearance and quality, such as identifying carrot root, green head, and crack. The traditional machine learning method realizes the classification of carrots through the extraction of feature points and color characteristics, which does not meet the requirement of real-time [13]. Deng et al. developed an automatic sorting system for external defects such as carrot cracks and fiber roots. In this study, convex polygon detection was used to detect carrot shape, concave points were adopted to verify fiber roots, and Hoff transformation was applied to detect carrot surface cracks [14]. Xie, Wang, and Yang et al. firstly extracted the features manually, and then the machine learning classifier SVM was selected to classify carrots [15]. Traditional machine learning algorithms require high image pre-processing and feature extraction, and they have poor strain on similar features. In addition, although traditional machine learning methods can provide better recognition of carrot defects, they generally suffer from the problem of large model size and are not suitable to be mounted on embedded devices of field machinery.

Based on the above problems, a deep learning network becomes important, which can automatically extract image features and solve various problems such as object classification, target detection, and segmentation, and is widely used in the field of intelligent agriculture. Chen et al. identified rice diseases by deep learning, with a recognition efficiency of 98.63% [16]. Jahanbakhshi et al. used the deep convolutional neural network to extract and classify the features of sour lemons with obvious surface defects, and the results showed that the classification accuracy of CNN for the sour lemons’ surface defect could reach 100% [17]. Zhang et al. utilized full convolutional networks (FCNs) to achieve accurate segmentation of internal bruises in blueberries, and its detection performance was significantly superior to SVM in terms of bruise detection [18]. Wang, Zhipeng et al. conducted a study on the real-time recognition of apple stem/calyx based on yolo-v5 algorithm [19]. The algorithm realized real-time detection at a speed of 25.51 frames/s with an accuracy of 93.89%. Deep learning also has many applications in carrot defect detection. Jahanbakhshi, Ahmad, et al. used deep neural networks to study the classification of carrot fruit shape. By improving the mean pool and maximum pool of the convolutional neural network (CNN), the accuracy of carrot classification was improved to 99.43% [20]. Zhu, Hongfei, et al. used a convolutional neural network to extract image features and support vector machines (SVM) for feature classification to achieve the sorting of carrot appearance quality [21]. Weijun Xie et al. combined deep learning with migration learning to identify the appearance defects of carrots, but the study could only identify carrots with clear defective features and was not applicable to the field environment [22]. Limiao Deng et al. achieved an automatic carrot grading process by using a deep learning approach, but the method had the problem of a large model size [23]. The realization of these models relies on datasets of high-resolution images. However, in the actual field combine harvesting, the fuselage jitter and uneven field road during machinery operation will lead to unstable shooting, insufficient lighting, and other problems, resulting in a low resolution of the carrot image, which is unfavorable for identification and sorting. The above studies mainly focus on pursuing higher accuracy, but there are few studies on the size and real-time performance of the model and a lack of analysis of the specific characteristics of the field environment, which is not suitable for carrying on the harvesters for field operation.

Knowledge distillation is a promising lightweight deep learning method. It aims to make the compressed, lightweight student network model have the same detection effect as the teacher network through the knowledge transmission of the teacher network with high performance. After knowledge distillation [24], lightweight student networks with high recognition performance are highly beneficial for deployment in embedded devices with constrained computing ability and mobile terminals. Li, Zhi, et al. proposed a multi-task knowledge distillation method based on mutual learning between image enhancement and semantic segmentation to achieve fast semantic segmentation of degraded images in bad weather. However, this method conducted data enhancement on images with degraded resolution to generate clear images and then classified the pixels, which did not learn the fuzzy features [25]. Wang Zhendong et al. applied the triple convolutional neural network for knowledge distillation to improve the detection speed of industrial CPS anomalies. However, this method needs to optimize a large number of hyperparameters and takes a long time for verification [26]. Most previous knowledge distillation studies focused on the lightweight design of the network, and there are few studied on the direct recognition of pixel degraded images, which were unsuitable for detecting carrot surface defects while operating a carrot combine harvester.

In conclusion, this study proposed a novel mechanism for carrot surface defect detection and sorting and innovated a multi-stage knowledge distillation model. A student network suitable for processing low-resolution carrot defect images was constructed, which greatly ensured the quality and food safety of carrots that flowed to the market. In order to adapt to the specific situation of carrot sorting during field harvesting, we adopted the improved knowledge distillation structure to guide the student network whose input end is a low-resolution image by using the teacher network whose input end is a high-resolution image [27]. The characteristics of different levels learned by the teacher network were assigned different weights to guide the lightweight student network in extracting discriminant features from fuzzy images [28,29]. Finally, the object detection network is established to recognize the low-resolution carrot image by means of an innovative knowledge distillation structure [30]. Simultaneously, to enable the constructed lightweight network to be carried on the intelligent terminal of the carrot combine harvester, we analyzed the effects of various student network structures on carrot defect detection and sorting and determined the most suitable lightweight network structure for field work. Finally, it is proved that the proposed algorithm is generalizable to all kinds of carrot defect targets through verification analysis.

## 2. Related Works

### 2.1. Lightweight Convolutional Neural Network

**Figure 1:**
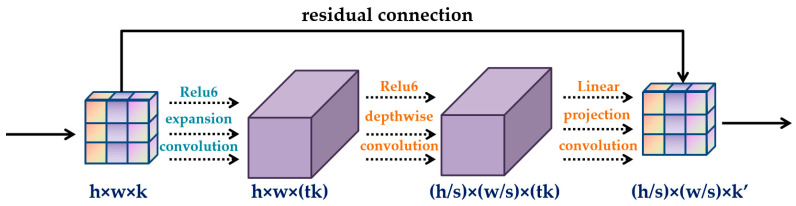
Basic structure of mobilenetV2.

An important means in improving the efficiency of the model is to design a more efficient lightweight network model without affecting the efficiency of model. At present, researchers have proposed various lightweight convolutional neural networks. ShuffleNet uses pointwise group convolution to reduce computing loss. The channels of each part of the feature map are shuffled by channel shuffle to form a new feature map. Ghostnet transforms a group of internal feature mappings into ghost feature mappings through the application of another linear transformation, which reflects the change information of internal features. The Mobilenet series network is a lightweight convolutional neural network with deep separable convolutions as the core. The deep separable convolution decomposes the standard convolution into two steps: the deep convolution and the pointwise convolution. Deep convolution first uses different convolution kernels to process multi-layer channels before using 1 × 1 pointwise convolutions to fuse the decomposed multiple channels. Traditional convolution operation requires im2col mode to change convolution operation into matrix multiplication. However, in mobilenet, the computing amount of 1 × 1 CONV exceeds 90.00%. As a kind of vector, it does not need the matrix acceleration operation, which greatly improves the computation ability of the model [31]. In addition, the mobilenetV2 adopted in this study utilizes a new activation function called relu6 to effectively reduce feature loss. The basic structure of mobilenetV2 is shown in Figure 1.

### 2.2. Object Detection

Finding the object of interest in the image and determining the category and location of the object is the core of the target detection task. There are several common methods of object detection. The R-CNN series is the first to use a preset anchor to determine the target object. The SSD is widely applied for small targets because of its pyramid structure for extracting features from feature maps of different scales. The yolo-v5 series is a collection of single-stage object detection methods that are composed of a backbone network, a detection neck, and three detection heads. At present, the yolo-v5 series is widely used in object detection. By adding an attention mechanism to the yolo-v5 network model, the overfitting of the model can be effectively reduced, and the generalization ability of the model can be improved, which is used for the identification of the tomato virus [32]. The combination of yolo-v5 and union non-maximum suppression distance intersection (DIOU-NMS) allows for the accurate counting of total wheatear and diseased wheatear [33]. This series can complete the feature analysis of all objects in the image. According to the different network depth and width settings, it can be divided into yolo-v5x, yolo-v5l, yolo-v5m and yolo-v5s, of which the yolo-v5s network has the smallest scale and the lowest accuracy but significant advantages in detection speed. The characteristics of the dataset in this study are that the positive sample area captured by the camera occupies a large proportion of the detection domain, which is typical for large-target detection. Meanwhile, in order to adapt and improve to real-time detection during the track transmission of harvester, we chose yolo-v5s [34].

### 2.3. Knowledge Distillation

Knowledge distillation is a model compression method that expects to obtain less parameter volume and computation amount; that is, the teacher network with more parameters assists the student network with fewer parameters to achieve better identification on the dataset through knowledge transfer. There are many categories of knowledge distillation. Fitnets primarily train a small student network using the output of the hidden layer in the middle. Its training result is obviously superior to the network model, whose knowledge output is the fully connected layer [35]. Attention can also be treated as a kind of knowledge and transferred to the student network [36]. However, the above studies are mainly aimed at the training of the same dataset, which does not consider that the resolution degradation of samples may exist in practical applications and cannot fully utilize the knowledge of the intermediate feature layer. To solve these problems, we propose a knowledge distillation model based on the idea of a “teacher-student network” which carries out two training steps. The teacher network is first trained on hig-resolution image datasets, and then the student network is trained on low-resolution image datasets. The teacher network has a more complex structure than the student network. The features extracted from the input image can be used to guide the student network and improve its feature identification ability [37].

The work of a carrot harvester is completed in a complex field environment. In order to achieve real-time and accurate sorting during the harvesting process, we decided to install a small and flexible intelligent terminal on the harvester. However, its computation ability is limited, and the number of network parameters trained by the yolo-v5s is relatively large, which makes it unsuitable for direct deployment. To achieve the balance between detection performance and model complexity, the knowledge distillation method is selected in this study [30].

## 3. Materials and Methods

### 3.1. Test Materials Acquisition

**Figure 2:**
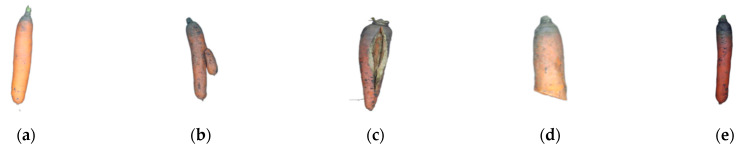
Part of experimental materials, (**a**) Normal; (**b**) Bifurcate; (**c**) Cracking; (**d**) Breakage; (**e**) Greenroot.

In this study, “Hongshen Qicun” carrots planted in Qingfeng Village, Harbin City, Heilongjiang Province, China (126°65′ E, 45°78′ N) were selected as the experimental material. The carrots have a fast growth rate, a high three-red rate, nearly cylindrical roots, and a uniform and beautiful appearance [38]. The collection period consisted of one harvest period. 5000 carrot samples were selected in total, and each data collection was finished on the day of harvest. The harvested carrots can be divided into five categories: normal, bifurcate, cracking, breakage, and green root. The length of the carrots ranged from 15 cm to 20 cm. Bifurcation occurs mainly because the carrots encountered rocks and other hard objects during growth, resulting in abnormal growth, appearance changes, and being more susceptible to mildew, reducing the edible value. The bacterial infection of carrots would cause cracking and breakage, thereby losing the edible value. Green root is produced by the chlorophyll on the surface of carrots when they are exposed to sunlight, which affects the taste [1]. The types of picked carrots are shown in Figure 2, and the details of picked carrots that will be harvested in October 2021 are listed in Table 1.

### 3.2. Dataset Construction

#### 3.2.1. System Design and Image Acquisition and Division

**Figure 3:**
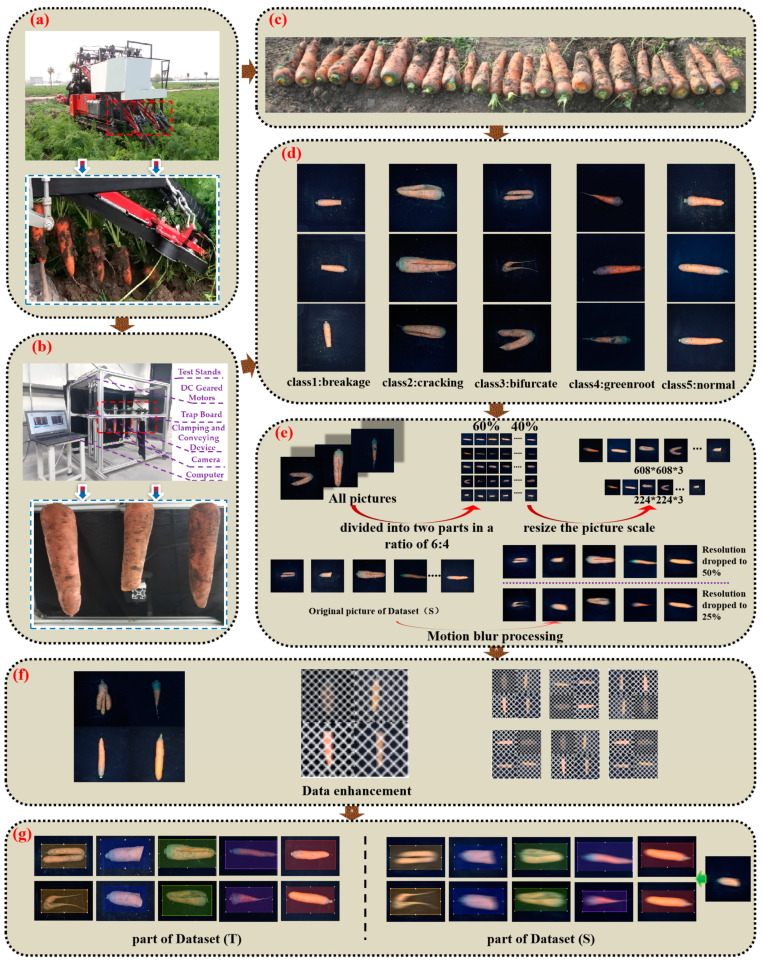
Acquisition of a carrot dataset. (**a**) Operation of the carrot combine harvester conveying system; (**b**) combine harvesting and collecting simulation system; (**c**) material acquisition for field tests; (**d**) classification of carrot defect types; (**e**) image preprocessing and dataset partitioning; (**f**) data enhancement; (**g**) partial dataset demonstration.

The surface defect detection and sorting system of the carrot combined harvester is composed of the combined harvest conveying system and a computer vision system. Figure 3a shows the operation of the conveying system of the carrot harvester. In order to ensure the authenticity of data collection, we built a simulation system of the combined harvest conveying, as shown in Figure 3b, which reproduces the combined carrot harvest environment. The aluminum alloy profile is the main body of the simulation system, and the limit plate is installed at the feeding part to ensure the same height of the carrot top during the feeding process. The clamping conveying device uses a 300 W DC reducer motor, which is connected to a DC motor governor to control the motor rotation speed so as to meet the requirements of the clamping conveying speed of 0.6 m/s–1 m/s in this study. Therefore, in this study, the clamping conveying speed was set to 0.6 m/s, and the distance between carrots during the image acquisition process was 100 mm. All sides of the platform were shielded by absorbing cloth to simulate the working environment of a combined carrot harvest. The computer vision system selected a Canon OM-M50 camera as the image acquisition device, with an effective pixel count of 24.1 million. In order to improve the integrity and flexibility of the sample image information, we applied two cameras and set the cameras to shoot at equal intervals according to the belt conveying speed and the distance between carrots. During the system testing stage, when there were any samples taken by one of the cameras that presented a defect, we identified the carrot sample as such a defect. The image processing algorithm was developed in Python 3.7 under the Windows 10 operating system, and PyTorch framework was selected. Hardware conditions are NVIDIA GeForce RTX 1650 TI GPU, Intel(R) Core(TM)i5-10400 CPU@2.90 GHz processor and 32 G memory.

In order to effectively extract the complete carrot surface features, a group of samples was rotated 90° after shooting to obtain the images of two other sides. The images from four different sides were obtained after the second shooting. The collected carrot images were cleaned and corrected, and a total of 16,000 carrot images meeting the requirements were retained, with 3200 original images for each type of carrot. A portion of the images obtained are shown in Figure 3d [31]. Two datasets were needed in this study, so the pictures we shot were divided into 6:4 by category, namely Dataset (T) and Dataset (S) respectively. Because the vibration of the engine and hydraulic pump led to the corresponding vibration of carrots combine harvester during operation, and the field land is not smooth, it causes blurring of the acquired images. In order to solve this problem, 576 images of each type were randomly extracted from Dataset (S) for motion blurring treatment using python language programming, of which 300 images of each type were processed with resolution reduction of 2 times and 276 images were processed with resolution reduction of 4 times. Dataset (T) is not resolution degraded. The specific process is shown in Figure 3e. The images in Dataset (T) and Dataset (S) were marked and enhanced with data to meet the requirements of subsequent neural network training.

#### 3.2.2. Data Preprocessing and Data Enhancement

In this study, Dataset (T) contains 9600 images, and Dataset (S) contains 6400 images. Dataset (T) and Dataset (S) data sets were divided into training set, verification set, and a test set, respectively. The ratio of image numbers in the three sets was 3:1:1, where degraded images of Dataset (S) with different resolutions should be randomly distributed in the training set, verification set, and test set. We used two training sets and two verification sets to train different models and tested the training performance of the models with test sets.

In order to facilitate subsequent image annotation and processing, we need to preprocess the image. Firstly, the weighted average method is used to calculate the grayscale of the color image. Due to the difference in height of the carrot before its is transmitted to the camera, there are two peaks of carrot tops and carrot in the gray histogram of some images. According to the needs of the experiment, we use threshold processing to extract the carrot as the region of interest (ROI), and the obtained ROI gray image and ROI color image are morphologically and mathematically calculated. Then we use the algorithm to resize the image to 608 by 608 pixels. Finally, Makesense.ai was used to label the five carrot types in the training sets for subsequent training (www.makesense.ai/).

In order to prevent the overfitting of the model, we conducted data enhancement on the above datasets to enhance the sample diversity, thus improving the generalization ability of the training model. Based on the features of the images taken, it was proposed to replace the original black occlusion area of two carrot surface defect datasets with white occlusion to increase the complexity of the model and generate regular oblique line segments with a certain distance between segments. The images were covered in interleaving ways, as shown in Formulas (1) and (2), and small features were retained. Finally, the two datasets were spliced in equal proportions, and the four enhanced images were randomly selected and spliced into one image in equal proportions, which was convenient for improving the training speed. As shown in Figure 3f.

*G*_x_, *G*_y_ = *random* (*G*_min_, *G*_max_) (1)

*W*_x_, *W*_y_ = *random* (*W*_min_, *W*_max_) (2)

where *G*_x_, *G*_y_, *W*_x_, and *W*_y_ are the distance and width of line segments in the horizontal and vertical directions.

### 3.3. Improved Multi-Stage Knowledge Distillation Carrot Surface Defect Detection Network

In the actual complex environment of farmland, the stability of communication signals cannot be guaranteed, so the client-cloud model cannot be well applied in field operations. The equipment performance of intelligent terminals is not as powerful as that of cloud servers, and the computing ability is limited. However, in order to realize the independent computation and guidance of intelligent terminals in the field, this study constructed an innovative knowledge distillation method to realize the sorting and detection of carrots during combine harvesting [29].

**Figure 4:**
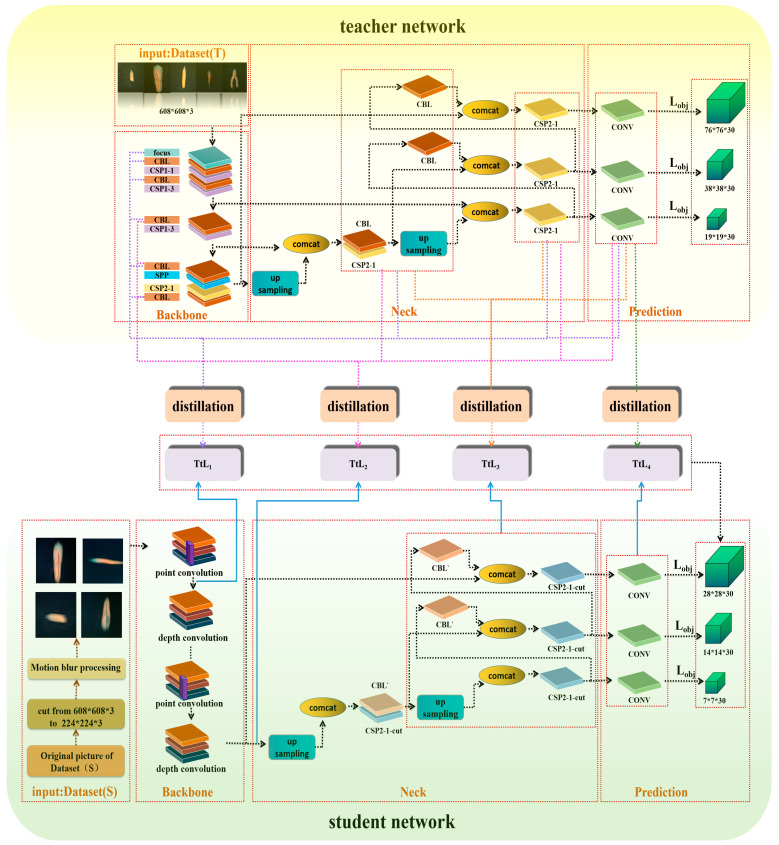
The main structure of the knowledge distillation training student network proposed in this study.

The whole knowledge distillation network is divided into a teacher network and a student network. First, yolo-v5s was adopted as the teacher network which was trained by Dataset (T) constructed in 3.2. We changed the backbone network of yolo-v5s to the lightweight mobilenetv2, which made the number of network layers go from 283 to 340 but decreased the number of participants compared with the teacher network. At this time, the model was already an excellent lightweight network, but to improve loading on the embedded devices, we pruned the network again to get the final student network. The Dataset (S) in 3.2 was used to train the student network. To improve the teacher network’s teaching effect on the student network and make knowledge present as a multi-stage expression, the knowledge output of yolo-v5s learning needed to be treated by a pyramid pool, so that the multi-layer features of the teacher network guided the single-layer features of the student network. The network structure is presented in Figure 4. The whole process is described in detail below.

#### 3.3.1. Improvement of the Student Network

The Student Network (mobile-slimv5s) takes yolo-v5s as the basis for improvement. In order to achieve effective loading of intelligent terminals in the field, we need to reduce the parameter scale of the student network as much as possible. Based on this problem, this study makes two improvements to yolo-v5s. First, the backbone network is changed by mobilenetv2, and secondly, the network of yolo-v5s is pruned to achieve higher model efficiency.

**Figure 5:**
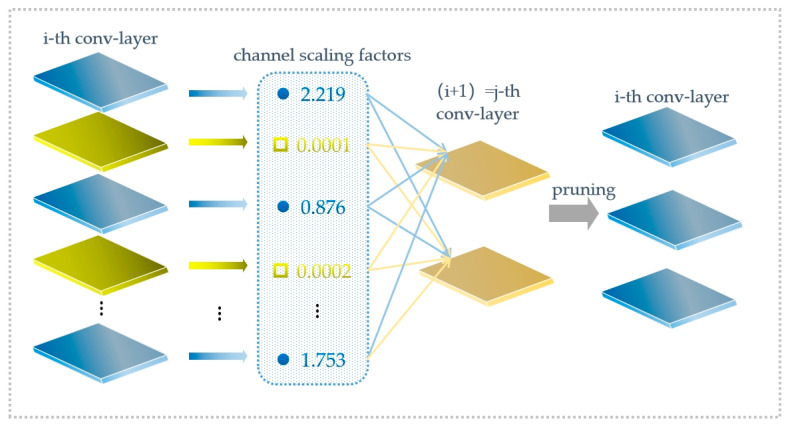
Process of network pruning.

The backbone network of yolo-v5s has a large number of parameters, so we choose the lighter feature extraction network, mobilenetv2. In order to make the changed network adaptable to yolo-v5s, the results of the last three down-samplings were output to yolo-v5s to complete the modification of the student network. After the replacement of the backbone network, this study adopts sparse training to prune the network of channel dimensions to further reduce the scale of the model. Since a carrot combine harvester is not suitable for hardware equipment with high computing efficiency when operating, the weighted sparsity is not suitable for this study, although it has better generalization ability and compression rate. The pruning at the network layer will cause a mass loss of feature information, which is not appropriate for the dataset with such a low resolution. The network pruning at channel level is a balance between the two. The channel pruning process is shown in Figure 5 [39].

In the whole network of yolo-v5s, the BN layer plays a role in improving model generalization ability and convergence speed. By adjusting the scaling factor of the BN layer in mobilenetv2 and the neck network, the sparsity excitation normalization of each channel in the BN layer is completed. The calculation of the BN layer is calculated by Formula (3). $Z_{in}$ and $Z_{out}$ represent the input and output of each BN layer, respectively. *γ* is the scaling parameter of the affine transformation, and *β* is the translation parameter of the affine transformation.

(3) $$Z=\frac{Z_{in}-\mu_{\beta }}{\sqrt{\delta_{\beta }^{2}+\varepsilon }},\;z_{\mathrm{out}}=\gamma z+\beta$$

*γ* is the scaling factor, and when its value is close to 0, the activation value will also be close to 0. Therefore, the network pruning of channel dimension must remove the corresponding weight of the channel whose *γ* is close to 0. In this study, we set the global threshold at 80%. The mask of the BN layer is obtained according to the threshold. The network parameters and computation amount are reduced after the pruning operation. In the neck network, there exists the situation that two convolutional layers add together. We add them normally in order to align the improved network channels. Two convolutional layers need to be merged to complete the network reconstruction. In this study, the size of the improved student network becomes 1/5 of what it was before.

#### 3.3.2. Innovation of the Knowledge Distillation

The parameters of the improved student network are small, but the identification efficiency of defect features is reduced. In order to meet the requirement that the carrot image after motion deblurring processing performs well in a simple, structured student network, we design to connect the features of multiple teacher streams before knowledge transfer and calculate the importance of different feature information to set different weights accordingly, so as to complete the guidance for a single student stream. Thus, the lightweight student network can complete the detection of images with reduced resolution.

First of all, we set Dataset (T) and Dataset (S) as the inputs of the teacher network (yolo-v5s) and the student network (mobile-slimv5s) to obtain the teacher model and student model, respectively. During the training of the student network, the motion blurring treatment would make part of the feature information disappear, which led to the poor training effect of the student network. In order to realize the correct recognition of image with fuzzy defect features, mobile-slimv5s requires parameter optimization under the guidance of yolo-v5s.

Assuming that the first i layer subnetwork of teacher flow and student flow are represented by i(t) and i(s), respectively, i(t) extracts discriminant features from Dataset (T). With the deepening of the network, the extracted knowledge changes from simple knowledge to abstract knowledge. i(t) completes feature extraction in the four stages of teacher network. In the backbone stage, feature information after Focus and CBL (Conv, BN, and Leaky relu) is extracted, respectively. The convolution kernel size of CSP1_X of Backbone is 3 × 3, and the step size is 2, which can complete down-sampling. Therefore, i(t) can obtain feature information with a size of 608 × 608, 304 × 304, 152 × 152, 76 × 76, 38 × 38, and 19 × 19, respectively, during the Backbone stage. Neck network transmits semantic features by up-sampling, transmission, and fusion of high-level feature information through FPN from top to bottom. Meanwhile, two PAN structures convey strong localization features from bottom to bottom. The two structures are integrated with each other. During this process, i(t) learns the converged features of different backbone layers, which are then distilled together with the characteristics learned by Backbone to guide mobile-slimv5s. In the prediction phase, the last layer is learned.

The above-mentioned stream features of all stages are fused through distillation. During the distillation process, we learn the structure of SKNet, cascade different feature maps, and get the fused feature maps. Then vector Z is obtained through the average pooling layer and the full connection layer, in turn. By initializing two matrices, A and B, corresponding weight matrices a and b are generated, and the importance coefficient of the information is obtained. As shown in Formula (4), different weight matrices are multiplied and added together with feature mappings, and the obtained results are calculated with the i(s) extracted by mobile-slimv5s to calculate the loss function.

(4) $$a=\frac{e^{AZ}}{e^{AZ}+e^{BZ}},\;b=\frac{e^{BZ}}{e^{BZ}+e^{AZ}}$$

i(s) extracts features from Dataset (S) and matches the output of i(t). From the above description, we know that distillation on a single output feature is similar to learning the defect name of only one carrot sample, which lacks the constraints on the middle layer. In other words, the feature judged by the teacher network has not been learned, which makes it easy to produce overfitting. Therefore, we obtain the output feature knowledge, relation feature knowledge, and intermediate feature knowledge of the teacher network in different stages and then fuse the relation feature and intermediate feature with the corresponding i(s) by means of four average pooling layers of different scales to calculate the loss function TtL1~TtL4 in the network structure diagram. The formula of the loss function TtL1~TtL4 is defined as:

TtL*_i_* = L_2_ (ap_1_ (FM_t_, FM_s_) + L_2_ (ap_2_ (FM_t_, FM_s_)) + L_2_ (ap_3_ (FM_t_, FM_s_)) + L_2_ (ap_4_ (FM_t_, FM_s_)) (5)

Formula (5) is used to calculate TtL1, TtL2, TtL3, and TtL4 in the network diagram. L2 stands for L2 loss function. FMt contains feature map data from different network layers of yolo-v5s. FMs is the feature extracted by mobile-slimv5s. Let the obtained features enter the average pooling before calculating the loss function. The api (FMt, FMs) represents the four-scale average pooling of the features of the teacher network and the student network on the api scale. The result is then calculated by the L2 loss function. Formula (6) is the calculation formula for the total loss function. In addition to the multilevel loss function described above, Lobj with the same object loss as yolo-v5s should be added. By adjusting the proportion parameters *α*, *β*, *θ*, and *γ*, the minimum loss function of student network can be obtained as follows:

*L*_total_ = *α*TtL_1_ + *β*TtL_2_ + *θ*TtL_3_ + *γ*TtL_4_ + L*_obj_* (6)

### 3.4. Operation Flow

Step 1: High-resolution Dataset (T) is used to train teacher network yolo-v5s and conduct performance tests. Meanwhile, Dataset (T) is used as the input for yolo-v5m and yolo-v5l for training, and performance characteristics are compared with yolo-v5s.

Step 2: The backbone of the teacher network is replaced with shufflenetv2 and mobilenetv2. Mobilenetv3 and GhostNet are treated as classifiers for feature extraction and network pruning to obtain a student network with a simpler structure. Low resolution Dataset (S) are used to train the student network and obtain the relevant parameter information.

Step 3: Pyramid pooling is conducted on the trained teacher network, and multi-stage teacher flow is used to guide the student flow so that the student network can extract more abundant low-resolution features. We will set the multi-stage distillation loss function parameters as: *α* = 0.1, *β* = 0.2, *θ* = 0.5, *γ* = 0.2.

### 3.5. Evaluation

#### 3.5.1. Basic Indicators

As seen from Table 2, the following indicators are used to evaluate and compare the models in this experiment. True positive, false positive, true negative, and false negative are used to calculate the precision and recall. Sensitivity is the proportion of samples that are correctly classified (i.e., samples corresponding to the given class). Specificity is the proportion of negative samples that are correctly classified (i.e., samples not corresponding to the given class). Accuracy is the overall classification rate of the classifier. These criteria are calculated using equations [39].

#### 3.5.2. mAP

The P-R curve takes the recall rate as the horizontal coordinate and the precision as the vertical coordinate. The curve formed on the basis of a certain threshold is called the P-R curve, and the area under the curve is the average precision (*AP*). The area under the PR curve is strictly *AP* curve. For multiple classification tasks, *mAP* is the arithmetic average of all *AP* classes.

(7) $$mAP=\frac{1}{a}\overset{a}{\underset{i=1}{\sum }}AP$$

## 4. Results

In order to evaluate the recognition performance of the network structure proposed in this study on the carrot dataset after motion blurring treatment, as well as the situation of a lightweight network, multiple sets of comparison tests were designed in the whole research process to select and verify the model, so as to realize the subsequent network loading on the intelligent terminal.

### 4.1. Effect of Teacher Network Scale on Distillation Effect

We selected yolo-v5 series with good stability in current object detection as the research object for the comparison of teacher network performance. In order to evaluate the influence of different teacher network scales on the performance of the student network, we first choose mobile-slimv5s as the student network. Then three networks, yolo-v5s, yolo-v5m, and yolo-v5l, were selected as teacher networks to evaluate their own training effects and supervision effects on student networks. We took Dataset (T) as input to train three teacher networks (T-yolo-v5s, T-yolo-v5m, and T-yolo-v5l) respectively, and then used Dataset (S) to train the improved lightweight network with/without teacher network supervision, so as to get S-mobile-slimv5s (with teacher network supervision) and mobile-slimv5s (without teacher network supervision). In the training of Dataset (T), the precision of carrot defect feature extraction increased with the increase in model complexity. The precision of yolo-v5s, yolo-v5m, and yolo-v5l is 0.736, 0.765, and 0.823, respectively. Yolo-v5l, with the highest model complexity, also has the best training effect. However, the number of parameters in yolo-v5l is significantly higher than the other two, and the calculation cost is higher.

We found that although their own precision was gradually improved, when they played the role of a teacher network to guide mobile-slimv5s, yolo-v5s had the best guidance effect. When the yolo-v5l was taken as teacher network to train Dataset (T), it presented obvious advantages, but its ability to supervise the student network was the worst. The experimental results are shown in Table 3. Under the supervision of yolo-v5s, the mAP of mobile-slimv5s is 0.879, an increase of 0.138 compared with 0.741 under the guidance of yolo-v5l, and increased by 0.156 compared with 0.723 under the guidance of yolo-v5m. Therefore, yolo-v5s is most suitable for the deployment of carrot defect sorting.

### 4.2. Effect of Student Network on Knowledge Distillation Effect

#### 4.2.1. Effect of Student Network Backbone

An appropriate backbone network can extract the features of positive samples quickly and accurately, reducing the complexity of the model. We selected the lightweight classification networks (shufflenetv2 [40], mobilenetv3 [41], GhostNet [42]) and the original darknet-53 as the backbone networks, respectively, and conducted the performance evaluation to compare with mobile-slimv5s after network pruning. Table 4 summarizes the performance of different networks. In experiment 4.1, we used yolo-v5s as a teacher network and trained it on Dataset (T) to obtain T-Yolov5s. The above-treated networks were regarded as student networks, respectively, and their performance on Dataset (S) was compared under the supervision of a teacher network. The results show that yolo-v5s, as a teacher network, has the highest precision, but its parameter numbers are almost 2.9 times those of mobile-slimv5s, 2.1 times those of shufflenetv2-yolo-v5s, and 2.24 times those of GhostNet-yolo-v5s. For the performance on Dataset (S), the accuracy of the mobilenet series is more than 90%, which is better than that of shufflenetv2 (88.3%) and GhostNet (82.9%). In order to balance computation and precision, Mobilenetv2 is more suitable for the backbone network of our student network [42].

#### 4.2.2. Effect of Student Network Model Hyperparameters

In this study, the control variable method is used to study the model hyperparameters, so as to find the most suitable dropout ratio, learning rate, and batch size of the student network.

- Effect of the dropout ratio

**Figure 6:**
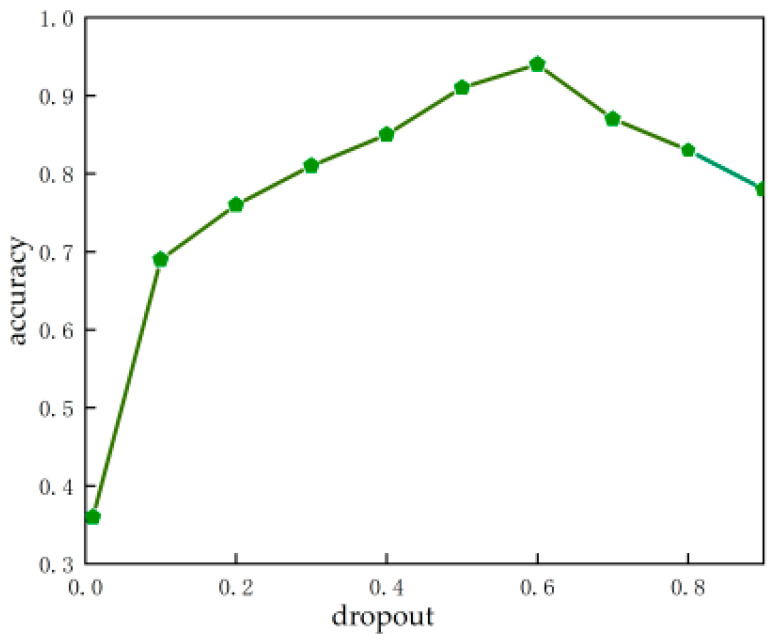
Effect of the dropout ratio on accuracy.

The dropout ratio represents the probability that the activation value of neurons in the network stops working, which can effectively avoid the model relying too much on some local features and improve the generalization ability of the model. The backbone structure of the S-mobile-slimv5s is mobilenetv2 [43], and the dropout ratio is added in the training. In this experiment, we studied the effect of different dropoust on model training in the optimal range of 0.01–0.9. Figure 6 shows the relationship between dropout and model accuracy [44]. From the figure, we can see that when the dropout increases from 0.01 to 0.6, the accuracy of the model also keeps increasing. From 0.6 onward, the accuracy slowly decreases as the dropout increases. When the dropout is too high, it means that too much neuron activity is suppressed, resulting in insufficient available features and reducing the accuracy of the model. The experiment indicates that suitable dropout can effectively suppress the overfitting of a model. In this study, the model shows the best performance when the dropout is 0.6.

- Effects of the learning rate

**Figure 7:**
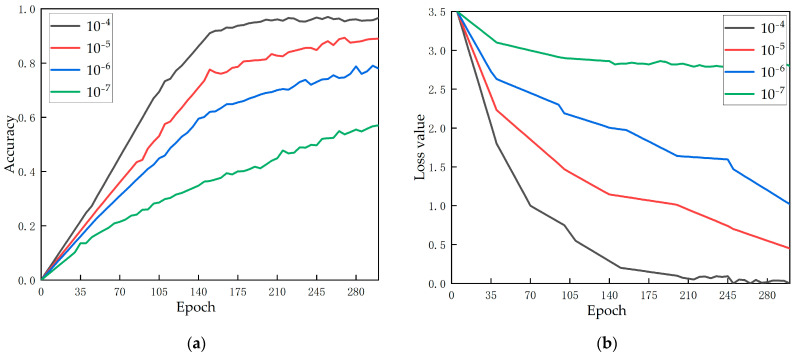
(**a**) Effect of the learning rate on accuracy; (**b**) Effect of the learning rate on loss.

The learning rate is used to adjust the weight of the network by controlling the step size of each iteration, so as to realize the hyperparameter of the optimal solution of loss function convergence. Figure 7 demonstrates the change of accuracy and loss value as the model training epoch lengthens under four different learning rates ($10^{-4}–10^{-7}$). As can be seen from Figure 7a, when the learning rate is $10^{-4}$, the accuracy is the highest; when the learning rate is reduced to $10^{-7}$, the accuracy increases slowly, and the training effect is not good. Within this range, the training time for the model to achieve optimal results decreases with an increasing learning rate. Figure 7b shows the loss of function’s variation with the learning rate. When the learning rate is $10^{-7}$, the loss rate is relatively high. When the learning rate is $10^{-4}$, the loss rate is the smallest. In the range of $10^{-7}$ to $10^{-4}$, the loss rate decreases with the increase of the learning rate. In this study, the model shows the best performance when the learning rate is $10^{-4}$.

- Effect of the batch size

**Figure 8:**
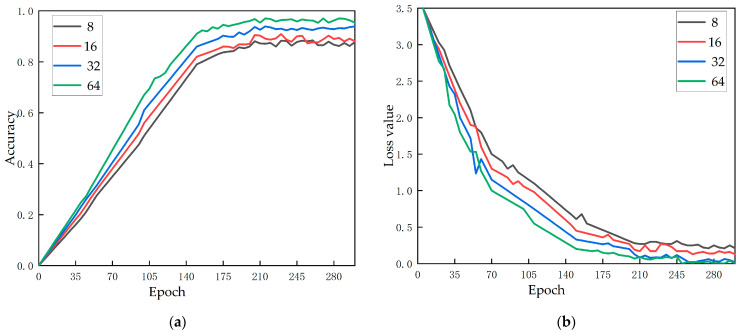
(**a**) Effect of the batch size on accuracy; (**b**) Effect of the batch size on loss.

The batch size refers to the number of images trained in each batch during the training process, which determines the training speed and time of the model and is affected by the model size, image batch, and hardware equipment of network training. When the batch size is set too large, exceeding the acceptable range of the computer, it will cause a shortage of memory, resulting in the termination of training. If the batch size setting is too small, the convergence speed of the model will be too slow, and the training time will be extended. Therefore, within the acceptable range of computer performance, we set the batch size for the experiment from 8–64 for the experiment. The experimental results are shown in Figure 8. With the increase of batch size, the accuracy is obviously rising, and the loss rate is declining. Therefore, the batch size was set to 64 in this study.

### 4.3. Effectiveness Evaluation of Knowledge Distillation

#### 4.3.1. Comparative Test on the Effectiveness of Multi-Stage Knowledge Distillation

Table 5 shows the performance comparison of mobile-slimv5s under the two learning situations on Dataset (T), with or without teacher network supervision. Table 6 shows the performance comparison of mobile-slimv5s on Dataset (S) with/without teacher network supervision. The yolo-v5s and the improved student network mobile-slimv5s are trained on Dataset (T) and Dataset (S) datasets, respectively. Other settings, parameters, and test sets are the same. In the high-resolution dataset, mAP@0.50 of yolo-v5s is 90.9%, mAP@0.50 of the supervised S-mobile-slimv5s network is 88.1%, and mAP@0.50 of the unsupervised mobile-slimv5s network is 76.2%, which presents an obvious decreasing relationship (Table 5). In terms of the training on Dataset (S), the performance of supervised learning is obviously better than that of unsupervised learning. Meanwhile, compared with the performance on Dataset (T), the gap between T-Yolov5s and S-mobile-slimv5s is much smaller (Table 6), which indicates that the knowledge distillation network constructed in this study is effective in identifying carrot images with motion blurring features. Under the guidance of yolo-v5s, mobile-slimv5s can well learn key features from resolution-degraded datasets, which is related to multi-stage feature extraction.

**Figure 9:**
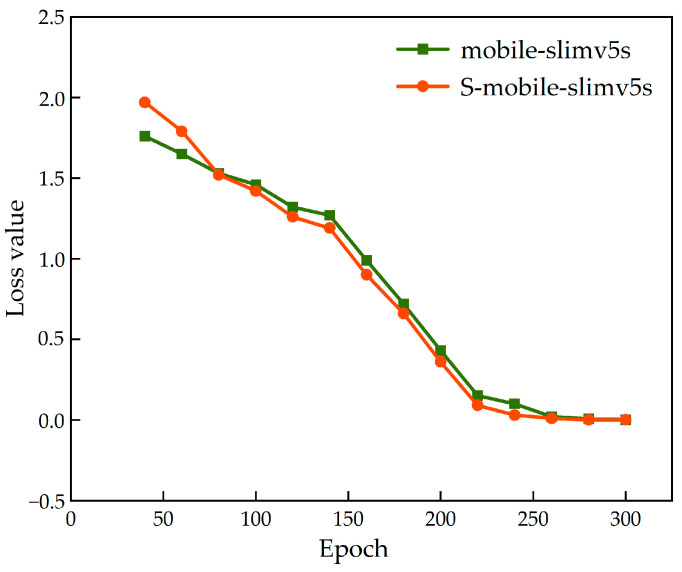
Comparison of loss value reduction of mobile-slimv5s with/without teacher network supervision.

Figure 9 shows the change in loss value of the teacher network (T-yolo-v5s) and its supervised student network (mobile-slimv5s) during the training process. At the beginning of training, the loss value of S-mobile-slimv5s was higher than that of mobile-slimv5s. When the training is completed 80 times, the training results of student network with supervision gradually improve and eventually gachieve a lower loss value, which indicates that the feature extraction ability of the student network for low resolution image is gradually deepened under the supervision of the teacher network.

#### 4.3.2. Feature Extraction Visualization

**Figure 10:**
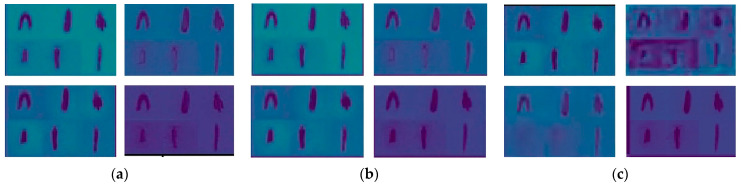
Model features visualization of part layers. (**a**) Features of the teacher network; (**b**) Features of student network with supervision; (**c**) Features of student network without supervision.

In order to observe the guiding effect of multi-stage knowledge distillation structure on the student network in Dataset (S) [45], output visualization of several network layers is carried out on the teacher network and the student network with/without teacher network supervision. The feature details for the visualization are shown in Figure 10. In this study, we set Dataset (T) as the input of the teacher network and Dataset (S) as the input of the student network. We could see that the outline of the target carrot in the feature map generated by the teacher network is clear and the noise is properly removed (Figure 10a). The student network has similar feature distribution under the guidance of the teacher network (Figure 10b). Figure 10c describes the student network that has not been supervised by the multi-stage features of the teacher network, so the feature map is quite different from that of the teacher network.

The experimental results show that the input of the student network is a low-resolution carrot image, but the student network can learn more details from the low-resolution carrot image and achieve a better identification effect through the guidance of the teacher network in the multi-stage knowledge distillation structure. Therefore, this study can effectively identify carrot defects after motion blurring treatment.

### 4.4. Overall Evaluation of Model Performance

**Figure 11:**
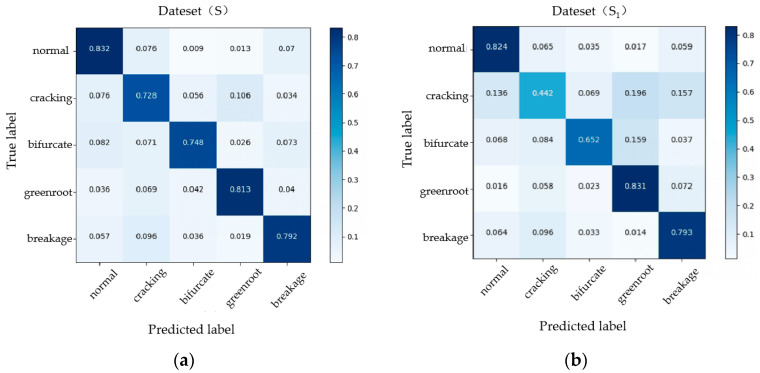
Confusion matrices of two different datasets (**a**) The confusion matrix of mobile-slimv5s when the input dataset is Dataset (S). (**b**) The confusion matrix of mobile-slimv5s when the input data set is Dataset (S_1_).

In order to verify the detection performance of network structure on different defect types of carrots, we tested four defect types in Dataset (S) and normal carrots [44]. Meanwhile, to fully verify the validity of this research, we carried out the same ratio of motion blurring treatment on the basis of Dataset (S) again to obtain Dataset (S_1_). The two datasets are compared, and the confusion matrix is shown in Figure 11. In the performance of Dataset (S), the accuracy of the algorithm in all defect types is higher than 70%, which meets the basic detection requirements. The detection effects on breakage and bifurcation are better, at 79.2% and 74.8%, respectively. The recognition accuracy of cracked, green root, and normal categories is 72.8%, 81.3%, and 83.2%, respectively. The cracking with defect features that are completely within the positive sample is the worst. In order to improve this error, we can increase the input proportion of cracking samples in the training set to improve the generalization ability of the network. The defect detection accuracy of Dataset (S_1_) with reduced resolution is 79.3% for breakage, 65.2% for bifurcation, 44.2% for cracking, 83.1% for green root, and 82.4% for normal. The distribution ratio is the same as that of Dataset (S).

**Figure 12:**
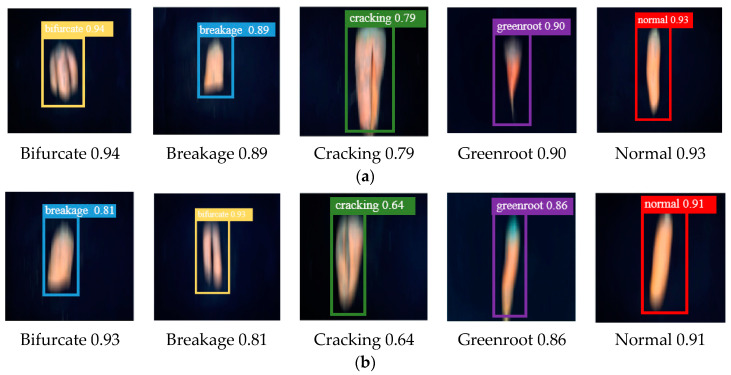
Identification result graph. (**a**) Part identification results of Datase t(S); (**b**) part identification results of Dataset (S_1_).

We present the test results of the model on two degraded datasets in Figure 12. The results show that our model has good detection performance. In the test phase, for several common carrot defect types, the student network can realize real-time detection without additional processing.

## 5. Discussion

The development of knowledge distillation provides a new solution for lightweight network design loaded on intelligent terminals, which solves the problem of the low feasibility of machine vision in many agricultural machinery operations. In this experiment, an improved multi-stage knowledge distillation method was used to supervise the designed lightweight network so as to identify the carrot surface defects. The performance of the teacher network is important for the training effectiveness of the student network [46]. In this study, we trained yolo-v5s, yolo-v5m, and yolo-v5l as teacher networks, respectively, but there were significant differences in the training results. This reason is that the structures of the three networks are similar, but yolo-v5s uses only one residual component (CSP1) that can increase the depth of the network, yolo-v5m uses two, and yolo-v5l uses three. CSP1 can increase the network depth and improve the abilities of feature extraction and feature fusion. The second difference is width_multiple, the network width parameter. The network width is controlled by the number of convolution kernels in different stages. The increase in the number of convolution kernels can improve the thickness of the feature map after convolution operation, thus improving the learning ability of network feature extraction. However, the results in 4.1 show that yolo-v5l, yolo-v5m, and yolo-v5s have decreasing supervision effects when they supervise the student network separately, in that order. Because S-mobile-slimv5s is used as a student network, its capacity is limited. So its ability to learn carrot defect features is also restricted. Excessive feature maps of yolo-v5l and yolo-v5m have become redundant information and have not been fully utilized. Secondly, the increment in model accuracy makes the hard label more significant, enhances the certainty of the data, and weakens the soft label. As a result, there is no suitable data to match the objects that complete knowledge transfer through soft label. At the same time, yolo-v5s is consistent with the baseline of the student network, which can reduce the errors in the process of matching the features of student network after the feature fusion.

This study meets the real-time requirement of carrot defect detection by replacing the backbone network of an ordinary neural network with a lightweight network with superior performance. [47]. Lightweight networks are already widely used in real-life applications. The integration model of MobileNetV2 and Xception can realize the fast prediction of plant diseases [48]. By improving the activation function of shufflenetv2 and adding the attention mechanism, fast garbage classification can be realized [49]. By using the search function of GhostNet and improving yolov4, pigeon behavior detection can be realized [50]. When considering the actual problem of surface defect detection during carrot combine harvesting in the field environment, different backbone networks of student networks need to consider real-time and accuracy simultaneously [51]. The results in 4.2.1 show that the student network has the highest accuracy when it is mobilenetv3-yolo-v5, but mobilenetv2 is superior in balancing model size and recognition efficiency. In general, shufflenetv2 performs better on open-source datasets. However, this study selected Dataset (S) as the dataset for training, which could better simulate the vibration of carrot harvesting machinery and resolution decline caused by too fast track transmission. Mobilenetv2 performs better in this dataset. The resolution reduction will result in the loss of pixel, texture, and other feature information. Mobilenetv2 first uses a 1 × 1 pointwise convolution operation and then raises the dimension six times to achieve depthwise convolution in a 3 × 3 high-dimensional space. Finally, by means of linear dimension reduction, the reversibility of the feature map is achieved to avoid the loss of important feature information of carrot defect during the activation of relu 6. Mobiletnetv3 reduces the number of convolution kernels in the first layer of mobilenetv2 from 32 to 16. Although this operation effectively reduces the number of parameters, fine-grained details will be lost when image features are difficult to capture, resulting in reduced accuracy. Mobiletnetv3 introduces the SE attention module on the basis of mobilenetv2 and uses h-swish activation function, which improves the capability of the degraded dataset to capture feature information. Therefore, in terms of the performance in Dataset (S), the accuracy of mobiletnetv3 is 0.2% higher than that of mobilenetv2, but there is no significant advantage. Meanwhile, the addition of the SE module leads to an increase in the number of mobiletnetv3 parameters. Compared with the mobilenet series, shufflenetv2 effectively reduces memory access by ensuring consistency between the input channel and the output channel, which is of great significance for intelligent terminal deployment. Shufflenetv2 replaces common convolution with pointwise group convolution and channel shuffle to improve the ability of feature extraction. However, mobilenetv2, which does not cause feature loss, has more advantages in Dataset (S). At the same time, the advantage of GhostNet network structure makes it have good performance in both accuracy and parameter scale on the dataset with reduced resolution. Therefore, mobilenetv2 was used as the backbone network in our study.

Knowledge distillation is an important means to improve the accuracy of the lightweight network model. Md ShakibKhan et al. used ResNet-50 as the teacher model and constructed the lightweight network DSNet as the student model to detect melanoma [52]. Through knowledge distillation, accurate recognition of low-light images can be achieved in lightweight networks with fewer parameters without increasing the computational burden of the model [53]. However, the previous knowledge distillation structure for crop recognition was mainly based on the learning of the full-connection layer (response-ased) or the middle feature part (feature-based). Compared with other knowledge distillation algorithms, the method proposed in this study has more advantages for degraded datasets. Whereas, response-based knowledge distillation or feature-based knowledge distillation are more suitable for supervised learning of high-resolution images [54]. Objects in high-resolution images have distinct features, so the distillation of a fully connected layer can get good results. However, in Dataset (S) with degraded resolution, the gray difference between pixels is small, and a single stage of knowledge output cannot express all features, so our multi-stage knowledge distillation shows its advantages. Due to the different characteristics of datasets, the discriminant features extracted by the teacher network in Dataset (T) do not all have positive effects on the students’ flow, which is the main reason why feature-based knowledge distillation could not get good performance in Dataset (T). Our structure innovatively converges features of different stages through average pooling. After multi-scale feature regression loss calculations, feature layers with different resolutions can be effectively fused. The important feature information is assigned a bigger weight, and the student flow is fine-tuned by the teacher flow through feature regression. Finally, mobile-slimv5s, which is supervised by the teacher network, can well adapt to carrot defect recognition after resolution degradation.

**Figure 13:**
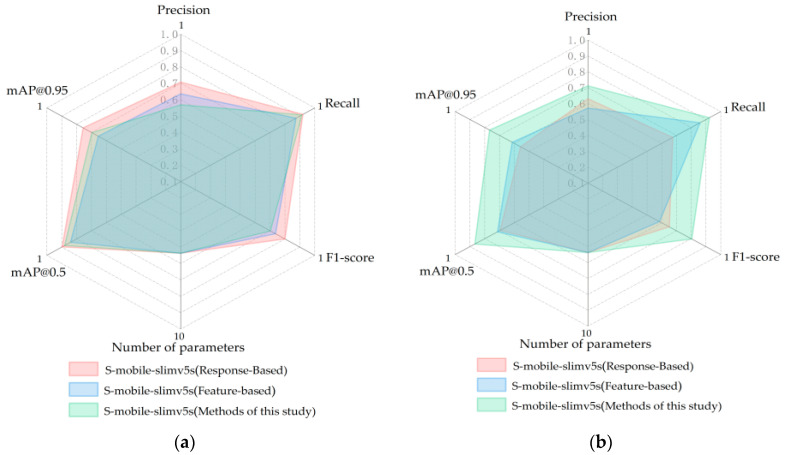
Performance of three knowledge distillation methods. Input data as (**a**) Dataset (T) and (**b**) Dataset (S).

In order to realize the correct recognition of carrot defect features under fuzzy conditions, this study proposed a multi-stage knowledge distillation that could adjust the weight ratio of feature-based and response-based and integrate them. The parameters are set to *α* = 0.1, *β* = 0.2, *θ* = 0.5, *γ* = 0.2. Since most of the previous studies were limited to high-resolution images with clear crop features, the previous knowledge distillation structure for crop recognition was mainly response-based or feature-based learning. In order to study the detection effect of our proposed knowledge distillation structure on the dataset with carrot resolution degradation, we compared the network results of this study with the response-Based knowledge distillation network and the feature-based knowledge distillation network. We applied three different distillation methods to train and guide the student network on the original dataset Dataset (T) and the degraded dataset Dataset (S), and compared their performance. The results are shown in Figure 13. In Dataset (T), the accuracy of feature-based reaches 96.3%, which is significantly higher than that of response-based (91.4%) and that of this study (92.6%). In high-resolution datasets, our supervision method does not show advantages. However, in the low-resolution Dataset (S), the test accuracy of our distillation method is 90.7%, only 1.9% lower than that in Dataset (T), while response-based and feature-based methods are reduced by 8.2% and 7.9%, respectively. Compared with the other two distillation structures, our structure can better extract the fuzzy features of carrots, least affected by pixel degradation [46]. In terms of parameter scale, our model is only 5.37 M. The more lightweight features make it more convenient to build intelligent terminals. 

The above research shows that the improved knowledge distillation method is feasible for defects sorting in the operation of combine harvesters. However, the principle and model proposed in this study are not suitable for the accurate classification of carrot images with normal surfaces. The category classification of a degraded dataset of normal carrot is the next research topic, which contains related issues of hyperspectral image classification. This is very meaningful research.

## 6. Conclusions

This study proposed a multi-stage knowledge distillation network. By building the student network in embedded sensors, the defect sorting of the carrot combined harvester in the field operation was realized. First of all, the yolo-v5s is lighter. We changed the backbone network of yolo-v5s to a more lightweight network and obtained the lightweight network mobile-slimv5s by pruning the network of yolo-v5s. The lightweight network model is only 5.37M, which fully adapts to the loading requirements of intelligent terminals. Secondly, this study adopts an innovative multi-stage knowledge distillation approach to supervised learning of student networks by T-yolo-v5s, which leads to effective recognition of carrot images with fuzzy features by student networks with an accuracy of 90.7%. It can meet the requirements for combined harvesting of carrots in the field. Finally, the carrot degraded images constructed in this study can be widely used for machine vision pre-training during the operation of other agricultural implements.

The algorithm proposed in this study is also applicable to the sorting of other fruits and vegetables after harvesting and can realize the simultaneous picking and sorting of a variety of cash crops during the harvesting process. It has important reference significance for improving the quick detection and sorting of scars and defective products. In the future, multi-spectral and infrared thermal imaging technology can be added to detect the internal conditions of crops and further improve the reliability of detection. In addition, the existing distillation method can be improved concentrating on the characteristics susceptible to blurring to improve the accuracy of sorting.

## Figures and Tables

**Table 1:** The categories of carrot: Normal; Bifurcate; Cracking; Breakage, and Greenroot.

No.	Classes	Quantity of Carrots
1	Normal	1138
2	Bifurcate	909
3	Cracking	964
4	Breakage	942
5	Greenroot	1047

**Table 2:** Evaluation indicators related to TP, FP, TN, and FN.

Evaluation Metric	Calculation Formula
Precision	$\frac{TP}{TP+FP}$
Recall	$\frac{TP}{TP+FN}$
Accuracy	$\frac{TP+TN}{TP+FP+TN+FN}$
F1-Measure	$\frac{2\cdot Precision\cdot Recall}{Precision+Recall}$
Specificity	$\frac{TN}{TN+FP}$

**Table 3:** Comparison of supervision effects under different teacher networks.

Teacher Network	Model	Precision	Recall	F1-Score	mAP@0.5	mAP@0.95	Number of Parameters (Million)
yolo-v5s as the teacher networks	T-yolo-v5s	0.736	0.946	0.828	0.909	0.827	15.36
	mobile-slimv5s	0.496	0.809	0.614	0.819	0.613	5.07
	S-mobile-slimv5s	0.512	0.923	0.659	0.879	0.766	5.37
yolo-v5m as the teacher networks	T-yolo-v5m	0.765	0.957	0.850	0.911	0.841	20.72
	mobile-slimv5s	0.496	0.809	0.614	0.819	0.613	5.07
	S-mobile-slimv5s	0.567	0.772	0.604	0.723	0.675	5.41
yolo-v5l as the teacher networks	T-yolo-v5l	0.823	0.972	0.891	0.932	0.867	49.9
	mobile-slimv5s	0.496	0.809	0.614	0.819	0.613	5.07
	S-mobile-slimv5s	0.517	0.714	0.599	0.741	0.701	5.46

**Table 4:** Performance comparisons.

Model	Precision	Recall	F1-Score	Accuracy	Number of Parameters (Million)	Loss (Epoch = 300)
T-yolo-v5s	0.736	0.946	0.828	0.976	15.36	0.00032
S-shufflenetv2-yolo-v5s	0.659	0.974	0.786	0.883	7.59	0.00016
S-mobile-slimv5s	0.512	0.923	0.659	0.901	5.37	0.00006
S-mobilenetv3-yolo-v5s	0.455	0.903	0.605	0.903	6.84	0.00013
S-GhostNet-yolo-v5s	0.536	0.917	0.677	0.829	6.85	0.00024

**Table 5:** Performance comparison of models when the input dataset is Dataset(T).

Model	mAP@0.50	mAP@0.95	Precision	Recall
T-yolo-v5s	0.909	0.827	0.736	0.946
mobile-slimv5s	0.762	0.764	0.581	0.857
S-mobile-slimv5s	0.881	0.793	0.569	0.915

**Table 6:** Performance comparison of models when the input dataset is Dataset(S).

Model	mAP@0.50	mAP@0.95	Precision	Recall
T-yolo-v5s	0.875	0.758	0.834	0.928
mobile-slimv5s	0.819	0.613	0.496	0.809
S-mobile-slimv5s	0.879	0.766	0.512	0.923

## Data Availability

Not applicable.
